# The light response in chickens divergently selected for feather pecking behavior reveals mechanistic insights towards psychiatric disorders

**DOI:** 10.1007/s11033-021-07111-4

**Published:** 2021-12-26

**Authors:** Clemens Falker-Gieske, Jörn Bennewitz, Jens Tetens

**Affiliations:** 1 Department of Animal Sciences, Georg-August-University, Burckhardtweg 2, 37077 Göttingen, Germany; 2 Institute of Animal Science, University of Hohenheim, Garbenstr. 17, 70599 Stuttgart, Germany; 3 Center for Integrated Breeding Research, Georg-August-University, Albrecht-Thaer-Weg 3, 37075 Göttingen, Germany; 4 Division of Functional Breeding, Department of Animal Sciences, Georg-August-Universität Göttingen, Burckhardtweg 2, 37077 Göttingen, Germany

**Keywords:** Feather pecking, GABA, Transcriptomics, Schizophrenia, Genome-wide association study

## Abstract

**Background:**

Feather pecking is a serious behavioral disorder in chickens that has a considerable impact on animal welfare and poses an economic burden for poultry farming. To study the underlying genetics of feather pecking animals were divergently selected for feather pecking over 15 generations based on estimated breeding values for the behavior.

**Methods and results:**

By characterizing the transcriptomes of whole brains isolated from high and low feather pecking chickens in response to light stimulation we discovered a putative dysregulation of micro RNA processing caused by a lack of *Dicer1*. This results in a prominent downregulation of the *GABRB2* gene and other GABA receptor transcripts, which might cause a constant high level of excitation in the brains of high feather pecking chickens. Moreover, our results point towards an increase in immune system-related transcripts that may be caused by higher interferon concentrations due to *Dicer1* downregulation.

**Conclusion:**

Based on our results, we conclude that feather pecking in chickens and schizophrenia in humans have numerous common features. For instance, a Dicer1 dependent disruption of miRNA biogenesis and the lack of *GABRB2* expression have been linked to schizophrenia pathogenesis. Furthermore, disturbed circadian rhythms and dysregulation of genes involved in the immune system are common features of both conditions.

**Supplementary Information:**

The online version contains supplementary material available at 10.1007/s11033-021-07111-4.

## Introduction

Feather pecking (FP) in chickens is a damaging obsessive behavioral disorder with a genetic component [1]. Common features with obsessive compulsive disorder like involvement of immune mechanisms have been reported [2]. Furthermore, in previous studies, we identified putative enhancer RNAs that target schizophrenia-associated genes [3] as well as numerous genetic variants in genes that have been previously linked to schizophrenia, namely *GABRB2*, *SPATS2L*, *ZEB2*, and *KCHN8* [4]. Hence, FP may be a potential model system for these conditions. A recent study reported major differences in the diurnal rhythm of gene expression between schizophrenia patients and healthy controls [5]. The study by Seney et al. revealed that healthy individuals and schizophrenia patients express two different sets of rhythmic transcripts and discovered an influence on GABAergic-related transcripts. This led us to reevaluate the brain transcriptome response of chickens divergently selected for high and low FP to light stimulation, a major trigger of FP behavior [6].

## Material and methods

All experimental procedures were described in a previous study [3]. Briefly, White Leghorn strains were selected for over 15 generations based on estimated breeding values for feather pecking. Rearing and husbandry conditions have been described by Bennewitz et al. [7]. At the age of 27 weeks, 48 hens (12 full-sib pairs from each strain) were phenotyped according to established protocols. Observation of feather pecking behavior was done in 20-min sessions on four consecutive days by a minimum of six different trained observers. To prevent FP birds were kept under low light conditions. One bird from each full-sib pair kept under dark conditions was sacrificed and whole brains were immediately collected for RNA isolation. Chickens were CO_2_-stunned and sacrificed by ventral neck cutting. For light stimulation, the remaining birds were kept under increased light intensity (≥ 100 lx) for several hours. Upon initiation of FP behavior these birds we sacrificed as well and brains were collected for RNA isolation. For the detection of genetic variation between the two chicken lines animals were phenotyped in groups of 42 hens at the age of 32 weeks and observed in 20 min sessions by seven independent trained observers [4]. Phenotypic values were standardized to 420 min observation time followed by box-cox transformation as described by Iffland et al. [8]. Analysis pipelines of transcriptomic and genomic data are outlined in our previous studies [3, 4]. Briefly, Illumina short RNA sequencing reads were trimmed and filtered with trimmomatic, mapped to the chicken reference assembly GRCg6a with TopHat, differential expression analysis was performed with DEseq2, and gene set enrichment analysis with clusterProfiler. Variant calling from genomic data was performed according to the GATK best practice guidelines. SNP chip data were imputed with Beagle and GWAS was conducted with gcta.

## Results

**Fig. 1:**
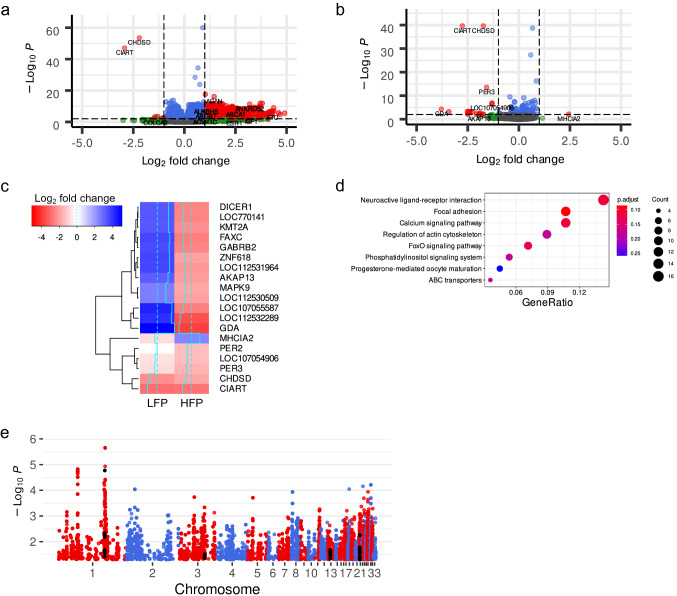
Volcano plots of differential gene expression in whole brains from **a** low feather pecking chickens and **b** high feather pecking chickens in response to a light stimulus. Grey dots represent transcripts that were not differentially expressed, green transcripts were above an absolute log_2_ fold change threshold of 1, blue transcripts were below an adjusted p-value of 0.01, and red transcripts were above an absolute log_2_ fold change threshold of 1 and were below an adjusted p-value of 0.01. Log_2_ fold change and adjusted p-values threshold are indicated by dashed lines. **c** Heatmap of log_2_ fold changes of genes differentially expressed in high feather peckers (HFP) in comparison to low feather peckers (LFP). **d** Gene cluster analysis results of KEGG pathways for genes differentially expressed in LFP in response to light. **e** Manhattan plot of GWAS hits with a p-value < 0.05 for the phenotype “feather pecks delivered cox-box transformed” performed on half-sibs convergently selected for feather pecking behavior. Variants in proximity to or located in genes coding for GABA receptors are shown in black

Low feather peckers (LFP) respond to light by upregulation of 714 and downregulation of 11 transcripts with 249 of these transcripts annotated as non-coding RNAs (ncRNAs). Surprisingly, high feather peckers (HFP) only show upregulation of one and downregulation of 18 transcripts (abs. log_2_ fold change > 1, adj. p-value < 0.01, Fig. 1a, b, Supplementary Information S1). To highlight the different directions of expression of a majority of these transcripts log_2_ fold changes of differentially expressed genes (DEGs) from the HFP group in comparison to the LFP group are shown in a heatmap (Fig. 1c). Significantly associated KEGG pathways after gene cluster analysis of DEGs in LFP brains in response to light compared to animals kept in the dark are shown in Fig. 1d to illustrate the loss of pathway activation in HFP (summary of results in Supplementary Information S2). Due to the low number of DEGs in HFP no gene cluster analysis could be performed. To identify genetic variation that might explain the strong difference between the two chicken lines a previously performed genome-wide association study (GWAS) [4] was repeated with a modified phenotype: feather pecks delivered box-cox transformed (Fig. 1e, Supplementary Information S3). We observed a strong peak on chromosome 1 that contains variants associated with *GABRA5* and *GABRG3*. Furthermore, we discovered GWAS hits (p-value < 0.05) on several chromosomes in proximity to or within the genes *GABRA1*, *GABRB2*, *GABRD*, *GABRG2*, *GABRG3*, *GABRR1*, and *GABRR2*. The functionally most interesting variant among those is rs733309797 on chromosome 13 at position 8,186,801 (p-value = 0.044), which was predicted to be a splice region variant in the *GABRB2* gene.

## Discussion

HFP exhibit a surprisingly low level of excitability to the light stimulus. An overall reduced variability of gene expression levels in whole brains of HFP was previously reported [9]. However, an even more remarkable difference between the two chicken lines was the direction of the log_2_ fold changes of DEGs in HFP (Fig. 1c). The majority of genes downregulated in HFP were upregulated in LFP in response to light. Since *Dicer1* is among those genes we hypothesize that the processing of and consequently the signaling by miRNAs is disturbed in HFP birds. Among DEGs in LFP brains after light stimulation, we identified about one-third to be ncRNAs, which we already observed by comparing brain transcriptomes of HFP with LFP [3]. We assume that in HFP ncRNAs are not properly processed due to the absence of the Dicer1 protein. Similar observations were made in transcriptome analyses of post mortem human brains of schizophrenia patients [10]. The authors hypothesized that these “psychiatric ncRNAs” might have an impact on local splicing events leading to transcriptome dysregulation. However, in a more recent study, the authors suggested that a Dicer1 dependent disruption of miRNA biogenesis may play a role in schizophrenia pathogenesis [11].

GABRB2 is an ionotropic type A γ-aminobutyric acid (GABA) receptor, which has been linked to schizophrenia in multiple studies (reviewed in [12]). Downregulation of multiple miRNAs has been shown to have an impact on GABRB2 protein levels in humans with internet gaming disorder [13]. Furthermore, it was recently shown in a murine knockout model that the lack of GABRB2 leads to various schizophrenia-like symptoms [14], which goes in line with our observations. We observed a downregulation of *GABRB2* in HFP in response to light, which is considered a major trigger of FP behavior. We hypothesize that lower expression levels of *GABRB2* in HFP brains (Fig. 1c) are caused by miRNA dysregulation, which ultimately leads to a disruption of GABA-mediated cellular ion influx. GABA is classified as the major inhibitory neurotransmitter, which might explain the low number of DE genes in the brains of HFP in response to light: A constant high level of excitation in neurons in the absence of inhibitory GABA signaling may not leave enough room for a response to be induced, even with the most basic stimuli. Furthermore, this high steady-state of excitation in HFP brains might provide an explanation for the behavior on the physiological level. In addition, the genes *GABRA2*, *GABRB2*, *GABRE*, and *GABRG3* were upregulated in the LFP’s response to light (Fig. 1a), which further indicates that there is a lack of GABA receptor upregulation in HFP. In one of our previous studies, an intron variant in the *GABRB2* gene was among the top variants associated with extreme FP [4]. This motivated us to repeat our GWAS on SNP chip genotypes imputed to whole-genome density of this half-sib population selected for high and low feather pecking [4] with a modified phenotype (feather pecks delivered box-cox transformed) as described by Iffland et al. [8]. Various variants associated with FP in the proximity to GABA receptors were discovered in that study with a medium density SNP chip based approach. We also discovered genetic variants located in or in close proximity to seven GABA receptor genes including *GABRB2* in whole genome sequence density genotypes (Fig. 1e). This and the fact that *GABRB2* is among the top candidates in our transcriptome studies and two independent GWAS approaches make GABAergic signaling one of the most promising research targets for future FP studies. It needs to be clarified in functional studies, whether GABA levels significantly differ in the two chicken lines and whether the administration of GABA leads to a reduction in feather pecking behavior. If our theory holds true further research should focus on the dissection of the genetics behind this GABA receptor dysregulation to develop new strategies in the breeding of egg-laying chickens to effectively select against the causative alleles.

The only upregulated gene in HFP after light stimulation was *MHCIA2*, which has a high similarity to human *HLA-C* (e-value = 9 × 10^–69^ as determined by NCBI protein BLAST). *HLA-C* is a risk factor for schizophrenia [15] that is interferon-inducible [16]. Since Dicer represses the interferon response [17], a lack of Dicer as observed in HFP may lead to activation of immune response genes—a connection that we and others previously established [3, 18, 19].

Another observation that caught our attention was the significant downregulation of the core circadian rhythm genes *PER2* and *PER3* [20] in HFP in response to light (Fig. 1c). Evidence that disturbances in circadian rhythms trigger severe psychiatric disease has been accumulating [21]. Various studies reported disturbed circadian rhythms in schizophrenia patients or model systems in connection to *PER2* and *PER3* expression or gene polymorphisms [22–25]. *PER3* in particular was linked to attention-deficit hyperactivity disorder [26, 27], which would comply with a hyperactivity disorder model of FP as proposed by Kjaer [28]. The brain transcriptome response of LFP to the light stimulus leads to an upregulation of numerous KEGG pathways (Fig. 1d), all of which have been linked to the circadian clock [29–36]. In HFP we observe a complete loss of gene activation regarding these KEGG pathways, which we conclude to be the result of the previously mentioned high level of constant neuronal excitation. If the neurons of HFP are on a constant high level of excitation the brain most likely does not respond to even basic stimuli.

## Conclusion

We currently believe that downregulation of *Dicer1* leads to a decrease in miRNA production and further downstream to downregulation of *GABRB2* and a lack of upregulation of *GABRA2*, *GABRE*, and *GABRG3*. This could result in high steady-state levels of neuronal excitation in HFP. Furthermore, *Dicer1* is a repressor of the interferon response and its downregulation might lead to higher interferon concentrations. Interferons are major signaling proteins that activate various immune response pathways which might explain the previously described increase in immune system-related genes in HFP. The functional validation of these findings could lead to the genetic dissection of feather pecking and build the basis for breeding against this damaging behavior. However, additional validation of these findings needs to be addressed in commercial flocks of egg laying chickens to exclude that these findings are limited to chickens selected for high feather pecking behavior. Due to the manifold commonalities with human psychiatric disorders, especially schizophrenia, chickens that have been selected for FP behavior over multiple generations might serve as a representative model for these conditions.

## Supplementary Information

Below is the link to the electronic supplementary material.

Supplementary Information S1: Differentially expressed genes in brains of low feather pecking and high feather pecking hens after light stimulation (abs. log2 fold change >1, adj. p-value < 0.01). (XLSX 73 kb)

Supplementary Information S2: ClusterProfiler KEGG pathway analysis results of differentially expressed genes from brains of low feather pecking hens after light stimulation. (XLSX 5 kb)

Supplementary Information S3: Genomic variants from genome wide association study of low and high feather peckers with the phenotype “feather pecks delivered box-cox transformed” (p-value < 0.05). (XLSX 18757 kb)

## Data Availability

All methods applied here have been outlined in previous studies [3, 4]. The raw RNA sequencing data has been deposited at the NCBI Sequence Read Archive (BioProject ID PRJNA656654) and the raw whole genome sequencing data as well (BioProject ID PRJNA664592).
